# Genome-Wide Identification of the *RALF* Gene Family and Expression Pattern Analysis in *Zea mays* (L.) under Abiotic Stresses

**DOI:** 10.3390/plants13202883

**Published:** 2024-10-15

**Authors:** Baoping Xue, Zicong Liang, Yue Liu, Dongyang Li, Chang Liu

**Affiliations:** 1 College of Agronomy, Shenyang Agriculture University, Shenyang 110866, China; 2 Department of Plant Sciences, College of Life Sciences, Wuhan University, Wuhan 430072, China; 3 Institute of Applied Ecology, Chinese Academy of Sciences, Shenyang 110866, China

**Keywords:** *ZmRALF* gene family, systematic evolution, expansion, expression pattern

## Abstract

Rapid Alkalization Factor (RALF) is a signaling molecule in plants that plays a crucial role in growth and development, reproductive processes, and responses to both biotic and abiotic stresses. Although RALF peptides have been characterized in *Arabidopsis* and rice, a comprehensive bioinformatics analysis of the *ZmRALF* gene family in maize is still lacking. In this study, we identified 20 *RALF* genes in the maize genome. Sequence alignment revealed significant structural variation among the *ZmRALF* family genes. Phylogenetic analysis indicates that RALF proteins from *Arabidopsis*, rice, and maize can be classified into four distinct clades. Duplication events suggest that the expansion of the *RALF* gene family in maize primarily relies on whole-genome duplication. *ZmRALF* genes are widely expressed across various tissues; *ZmRALF1/15/18/19* are highly expressed in roots, while *ZmRALF6/11/14/16* are predominantly expressed in anthers. RNA-seq and RT-qPCR demonstrated that the expression levels of *ZmRALF7*, *ZmRALF9*, and *ZmRALF13* were significantly up-regulated and down-regulated in response to PEG and NaCl stresses, respectively. Overall, our study provides new insights into the role of the *RALF* gene family in abiotic stress.

## 1. Introduction

Rapid Alkalinization Factor (RALF) represents a class of small peptides enriched in cysteine residues, with precursor peptides comprising 80 to 150 amino acids [1]. RALF is expressed in various tissues and organs in plants, playing a pivotal regulatory role in processes such as plant growth, development, and responses to environmental changes [2]. The discovery of RALF peptides was first reported in tobacco [3]. These peptides, with a molecular weight of only 5 kDa, induce a rapid increase in the pH of tobacco cell suspension cultures and activate downstream MAPK signaling pathways [3].

RALF peptides are widely distributed across plants, including bryophytes, ferns, gymnosperms, and dicotyledonous plants [4], such as *Arabidopsis*, *Brassica napus*, and rice [5,6,7]. In *Arabidopsis*, RALF members significantly affect plant growth, development, and immune responses. For instance, *RALF1/4/19/23* are involved in regulating cell elongation, plant immune responses, and double fertilization [8,9,10]. Furthermore, RALF is implicated in pollen tube growth and the processes of pollination and fertilization [8,11]. Additionally, RALF participates in plant immune responses [12]. The immune-eliciting function of RALF often depends on its receptor kinase FERONIA (FER); in *fer* mutants, the immune-eliciting function of *RALF22* is lost or attenuated [13]. Furthermore, RALF can collaborate with other plant cytokines to jointly regulate plant immune responses. For example, RALF22 amplifies the immune signal induced by Pep3 (another plant cytokine), thereby enhancing plant immunity [13].

RALF peptides play a crucial role in mediating responses to various abiotic stresses, including salt, drought, and heat stresses. Overexpression of *AtRALF1* in transgenic plants has been shown to enhance tolerance to salt stress [14], while *AtRALF8* is induced by drought stress [15]. Conversely, overexpression of *AtRALF22* exhibits reduced tolerance to salt stress [16]. In cotton, *RALF33* responds to both low-temperature and salt stress [17]. The knockout of *PpRALF1/2/3* genes in *Physcomitrella patens* has been found to enhance tolerance to paraquat and salt stresses [4]. Furthermore, overexpression of quinoa *RALF15* in *Arabidopsis* leads to a reduced tolerance to salt stress [7].

RALF peptides, upon binding to receptors located on the cytoplasmic membrane, form receptor–ligand complexes that regulate a diverse array of biological processes [11]. Catharanthus roseus receptor-like kinase 1-likes (CrRLK1Ls) are widely present in plants and typically encompass two extracellular Malectin domains, a transmembrane domain (TMD) and an intracellular kinase domain. Members of the CrRLK1Ls family, such as FER, ANX1/2, BUPS1/2, and THE1, perceive changes in the external environment through interactions with RALF peptides, thereby modulating plant growth, development, and stress responses [18]. For instance, the FER receptor kinase recognizes the AtRALF1 peptide to regulate root cell growth [19], whereas the THE1 receptor kinase senses the AtRALF34 peptide to control lateral root initiation [20]. Moreover, ZmFERL4 receptor kinase recognizes the *ZmRALF2* and *ZmRALF4* peptide to regulate cell wall integrity during pollen tube germination and growth [21].

Maize is a globally critical crop and a staple food source. Extreme temperature, drought, and high salt stresses affect approximately 90% of cultivable areas, leading to a 70% reduction in the yields of significant food crops, including maize, sorghum, rice, and wheat. Given the pivotal role of RALF in plant growth, development, and stress responses, elucidating its functions in food crops is essential for enhancing resistance to environmental stress. Although *RALF* gene families have been identified in various plants [5,6,7], the functions of *RALF* remain are poorly understood in maize, particularly in response to abiotic stresses. Consequently, this study aims to conduct a comprehensive bioinformatics analysis of the *RALF* gene family in maize, identifying functional genes to facilitate the development of strategies aimed at improving maize resilience and productivity. In this study, we identified 20 *ZmRALF* genes based on the maize genome. Subsequently, we performed a systematic bioinformatics analysis of the *ZmRALF* gene family, which included phylogenetic relationships, conserved protein motifs, gene structures, cis-acting elements, and expression levels in various tissues under different abiotic stresses. This comprehensive analysis provides valuable insights into the potential roles of *ZmRALF* genes.

## 2. Results

### 2.1. Whole-Genome Identification of RALF Genes in Maize

**Figure 1:**
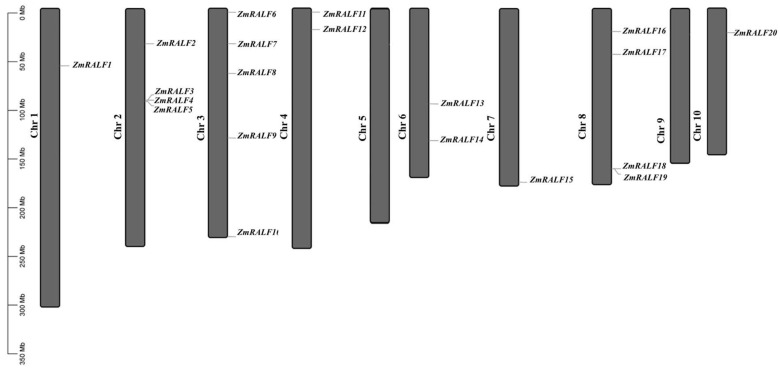
Chromosome distribution of *ZmRALF* genes. The distribution of 20 *ZmRALFs* genes on ten maize chromosomes.

RALFs are a class of multifunctional plant cytokines that can induce a rapid increase in the extracellular pH value of plants. RALFs are recognized by receptor-like kinase FER (FERONIA) and are involved in plant growth and development, cell elongation, stress response, immune response, and other life processes. To identify RALFs in the maize genome, we used BLASTP and HMM methods, and 20 *RALF* genes were identified from the maize genome (Supplemental Table S1). These *ZmRALF* genes are named *ZmRALF1*-*ZmRALF20* according to their location on the chromosome (Figure 1). Moreover, we analyzed the physical and chemical properties of RALFs. The amino acid lengths of these proteins range from 104 (ZmRALF10) to 145 (ZmRALF1) aa. The isoelectric point (pI) of ZmRALF family proteins varies from 4.63 to 11.28; specifically, ZmRALF1 and ZmRALF15 are classified as acidic proteins with a pI less than seven, while the remaining members are categorized as basic proteins with a pI greater than seven (Supplemental Table S1). The molecular weights of ZmRALF family proteins range from 10.79 to 15.04 kD. According to the grand average of hydropathicity analysis, ZmRALF1/3/4/8/10/17/18 proteins are classified as hydrophobic proteins, while the other members are considered hydrophilic proteins, exhibiting a grand average of hydropathicity less than zero.

### 2.2. Sequence Alignment and Phylogenetic Analysis of ZmRALF

**Figure 2:**
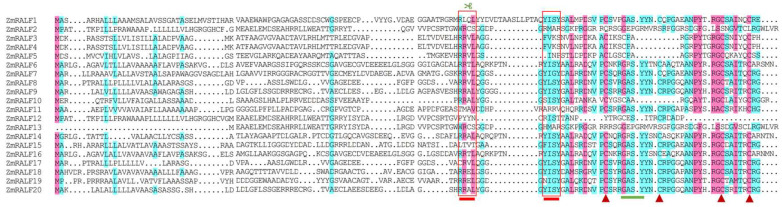
Multiple sequence alignment of ZmRALF proteins. Multiple sequence alignment was carried out using DNAman 8. The red triangles represent cysteine residues. The scissors represent the RRXL protease recognition site. The first red box represents the RRXL cleavage site and the second red box represents the YISY conserved motif. The blue lines represent GASYY conserved motif.

Previous studies have demonstrated that typical RALF peptides possess several conserved structures, including the RRXL protease recognition site, the YISY conserved motif, and four cysteine residues located at the C-terminus [18]. To investigate the conserved structure of maize RALF, we performed a sequence alignment of the RALF family proteins in maize. As shown in Figure 2, there are numerous structural variations among the RALF1/2/9/11/15/17/18/20 peptides, notably, the absence of RRXL cleavage sites, as well as incomplete cysteine residues and YISY motifs. In contrast, RALF3/5/8/12/13 exhibit a complete structure, including RRXL cleavage sites, YISY motifs, and four cysteine residues. Notably, RALF1 and RALF17 lack both RRXL cleavage sites and YISY motifs (Figure 2).

**Figure 3:**
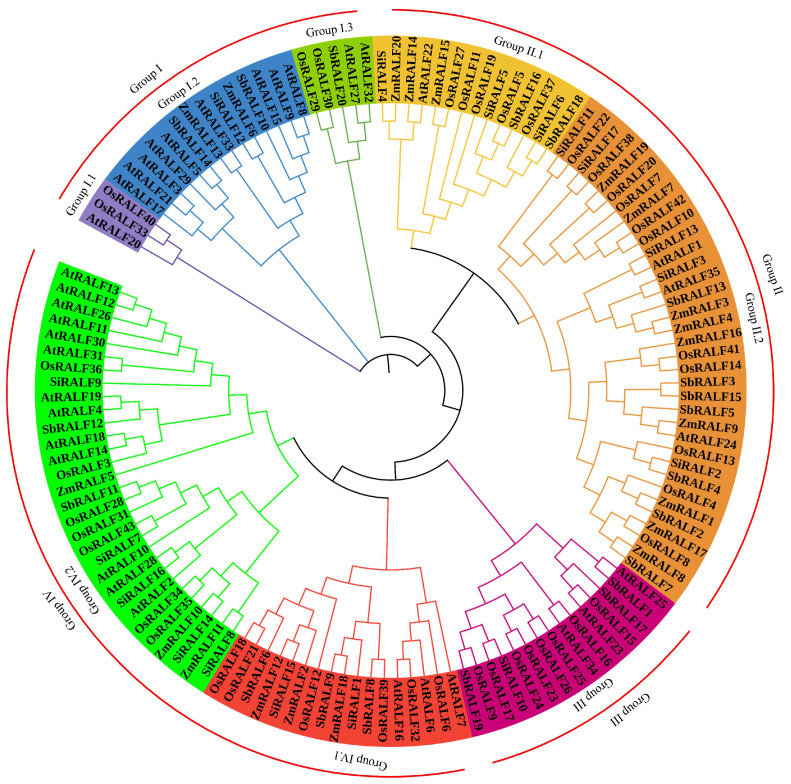
Phylogenetic tree of 133 proteins from ZmRALF, AtRALF, SiRALF, SbRALF, and OsRALF proteins. These protein sequences were aligned using MUSCLE, then a phylogenetic tree was constructed using the neighbor-joining (N-J) method with 1000 bootstraps in MEGA 7.0. The picture was created with the online tool iTOL 6.9.1 (https://itol.embl.de/, accessed on 20 July 2024). Different colors indicate different RALF subgroups.

To explore the evolutionary relationships among ZmRALF, OsRALF, and AtRALF proteins, we constructed a phylogenetic tree based on 20 ZmRALF, 41 OsRALF, 17 SiRALF, 20 SbRALF, and 35 AtRALF proteins using the neighbor-joining method of MEGA 7 software. Our analysis classified the 133 RALF proteins into four subfamilies: Group I, Group II, Group III, and Group IV. Notably, Group I is further subdivided into Group I.1, Group I.2, and Group I.3, while Group II is divided into Group II.1 and Group II.2, and Group IV is further divided into Group IV.1 and Group IV.2 (Figure 3). We observed that Group II.2 contained the highest number of family proteins, comprising 3 AtRALF, 11 OsRALF, 9 ZmRALF, 5 SiRALF, and 7 SbRALF proteins. In contrast, Group I.1 was the smallest subgroup, consisting of only one AtRALF and two OsRALF members. Remarkably, we found that no OsRALF was found in the Group I.2 clade, and no ZmRALF and SiRALF from maize and millet were identified in the Group I.3 clade, showing that Group I.2 and Group I.3 were lost in rice, maize and millet, respectively (Figure 3). Additionally, Group II and Group IV contained AtRALF, OsRALF, ZmRALF, SiRALF, and SbRALF members, suggesting that Group II and Group IV members are evolutionarily functionally conserved. This detailed phylogenetic analysis provides valuable insights into the functional diversity of RALF proteins in maize, millet, sorghum, rice, and *Arabidopsis*.

### 2.3. Gene Structure and Protein Conserved Motif of ZmRALF

**Figure 4:**
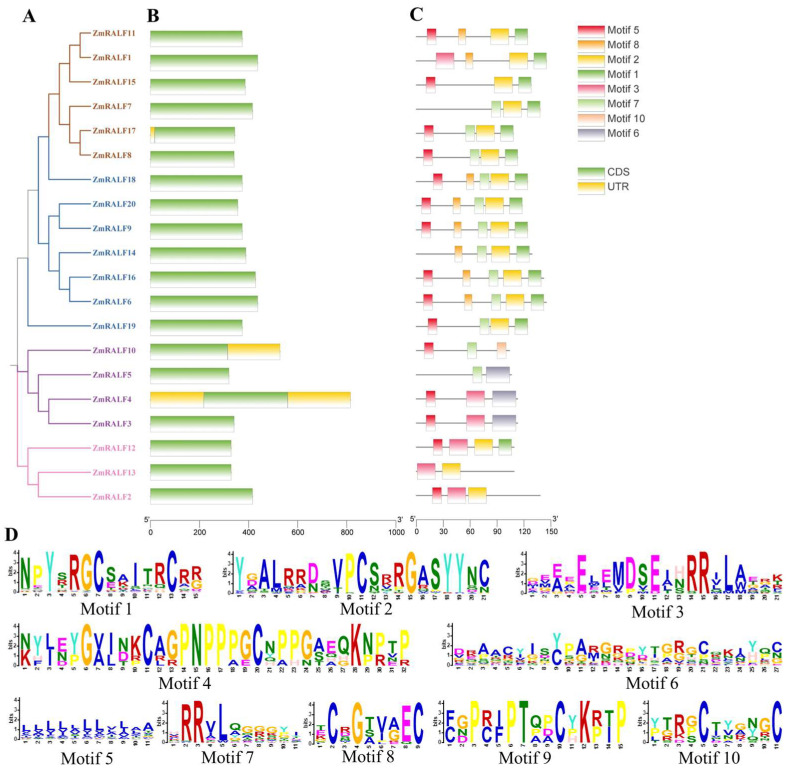
The gene structure and protein conserved motifs of ZmRALF. (**A**) The phylogenetic tree was constructed using MEGA 7.0 software (method: neighbor-joining; bootstrap: 1000). (**B**) The gene structure was analyzed using GFF files. (**C**) Protein conserved motifs were analyzed by MEME. (**D**) Protein sequence analysis of ZmRALF motifs.

The gene structure and the conserved motif play a critical role in the process of gene functional differentiation and evolution. To investigate the gene structure and the conserved motifs of RALFs in maize, we utilized the online website MEME and GFF files for our analysis. As shown in Figure 4, the gene structure analysis revealed that all members of the ZmRALF family are devoid of introns. This is consistent with previous studies on soybean, Medicago, and Lotus [22]. Notably, *ZmRALF4* possesses non-coding regions at both the 5′ and 3′ ends of the gene structure, while *ZmRALF17* and *ZmRALF10* exhibit non-coding regions at the N-terminal and C-terminal ends, respectively (Figure 4). Moreover, we conducted a conserved motif analysis using the online tool MEME, which revealed that RALF family members contain ten distinct motifs. Our findings indicate that motif 1 and motif 5 are present in the majority of ZmRALF family members. Specifically, motif 5 is absent in ZmRALF1, ZmRALF5, ZmRALF7, ZmRALF13, and ZmRALF14, while motif 2 is lacking in ZmRALF2, ZmRALF3, ZmRALF4, ZmRALF5, ZmRALF10, and ZmRALF13. Additionally, motif 6 and motif 3 are unique to ZmRALF5 and ZmRALF2, respectively (Figure 4). Protein sequence analysis of these motifs revealed that motif 1 contains the RCRR motif, while motif 2 contains the GASYY motif (Figure 4). The widespread presence of these motifs across the ZmRALF family suggests functional similarities among these members. Previous studies on soybean, Medicago, and Lotus demonstrated that the proportion of RR motifs were 83.33%, 66.67%, and 52.94%, respectively. Statistical analyses indicated that the proportion of RR motifs in maize was 65% [22]. In contrast, the proportion of RR motif in *Arabidopsis* is only 29.27% [22], suggesting that a significant number of RALF peptides in Arabidopsis cannot be processed to yield mature and active forms. Similar trends were observed in the statistical analysis of the four conserved cysteine residues. According to a previous study, cysteine residues at the C-terminal end can form disulfide bridges, which play an important role in the correct folding of the mature RALF protein [23]. The higher conservation of four cysteine residues in maize implied that ZmRALFs were more likely to form three-dimensional (3D) structures and to perform normal functions. This comprehensive motif analysis enhances our understanding of the structural and functional diversity within the ZmRALF gene family and provides a foundation for further functional characterization.

### 2.4. Homologous Gene Analysis of ZmRALF

**Figure 5:**
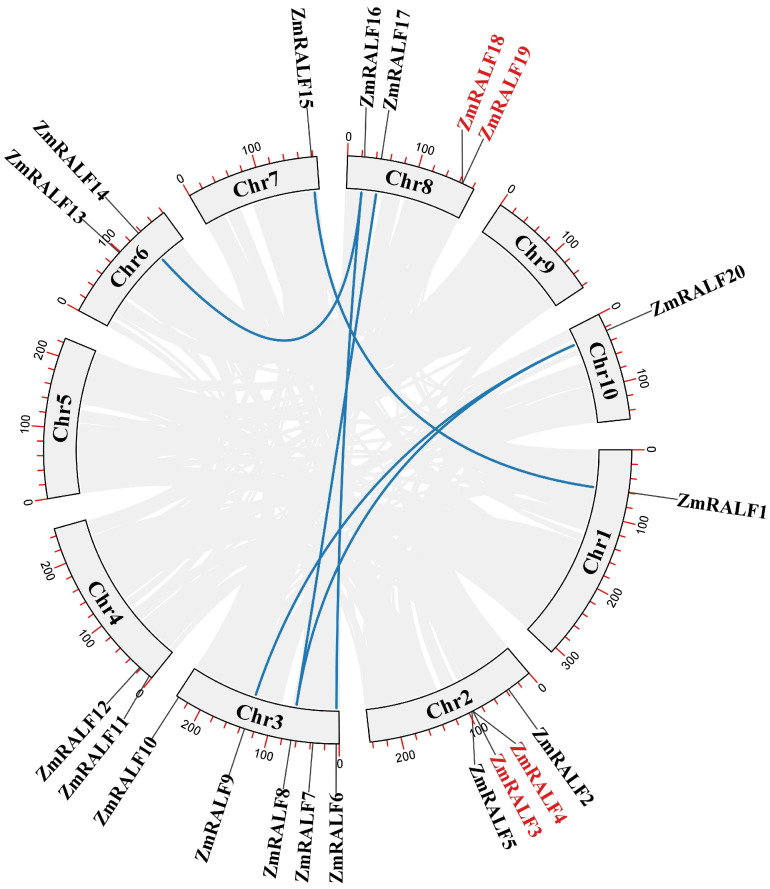
The duplication events analysis of *ZmRALF* genes. Names in red name represent tandem replication genes, while blue lines represent whole-genome duplication genes.

To investigate the expansion pattern of the *ZmRALF* gene family, we analyzed the duplication events associated with the *ZmRALF* genes. As depicted in Figure 5, we identified two tandem duplication gene pairs (four tandem duplication genes) and six whole-genome duplication gene pairs (twelve whole-genome duplication genes) in maize. Previous studies have indicated that the expansion of the *AtRALF* gene family is primarily attributed to tandem duplication [5], whereas the expansion of the *GmRALF* gene family mainly relies on whole-genome duplication [22]. Our findings reveal that the expansion pattern of the *ZmRALF* gene family resembles that of the *GmRALF* gene family, predominantly relying on whole-genome duplication. Furthermore, we analyzed the Ka/Ks values, as presented in Supplemental Table S2; most duplication *ZmRALF* gene pairs exhibited Ka/Ks values less than 1, with the exceptions of *ZmRALF1/ZmRALF11* and *ZmRALF4/ZmRALF18*, which displayed Ka/Ks values greater than 1. These results suggest that the *ZmRALF* gene family has undergone purifying selection during evolution. This comprehensive analysis of duplication events and Ka/Ks values offers significant insights into the evolutionary mechanisms driving the expansion of the *ZmRALF* gene family, underscoring the role of purifying selection in maintaining gene function.

### 2.5. Collinearity Analysis of ZmRALF Genes with OsRALF and AtRALF

**Figure 6:**
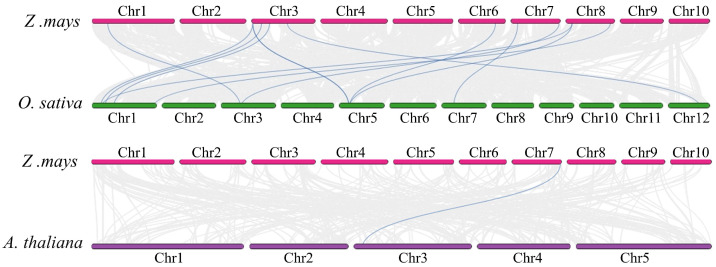
Collinear gene pair analysis of *ZmRALF* between maize and rice and *Arabidopsis*. The blue line represents collinear gene pairs. The pink color represents the maize chromosome, the green represents the rice chromosome, and the purple represents the *Arabidopsis* chromosome.

To further investigate the evolutionary mechanisms of the *ZmRALF* gene family, we analyzed the syntenic relationships between *ZmRALF* genes and their counterparts in rice (*OsRALF*) and Arabidopsis (*AtRALF*). As shown in Figure 6, we identified 13 syntenic gene pairs between maize and rice, as well as 1 syntenic gene pair between maize and *Arabidopsis*. This finding indicates that maize shares the most syntenic genes with rice, suggesting that the function of *ZmRALF* and *OsRALF* genes has been relatively conserved during evolution. In contrast, there appears to be significant functional differentiation between *ZmRALF* and *AtRALF* genes (Figure 6). Interestingly, *ZmRALF2* exhibited syntenic relationships within both rice and *Arabidopsis*, suggesting that *ZmRALF2* shares a common ancestor with these species. Furthermore, we discovered that three *ZmRALF* genes (*ZmRALF4*, *ZmRALF13*, and *ZmRALF18*) formed three syntenic gene pairs with one *OsRALF* gene (*OsRALF21*), while two *ZmRALF* genes (*ZmRALF2* and *ZmRALF11*) formed two syntenic gene pairs with another *OsRALF9* gene (Figure 6). These findings provide valuable insights into the evolutionary history and functional conservation of the *RALF* gene family across different species, highlighting the intricate syntenic relationships that have influenced their divergence and adaptation.

### 2.6. Analysis of Cis-Elements of ZmRALF Gene

**Figure 7:**
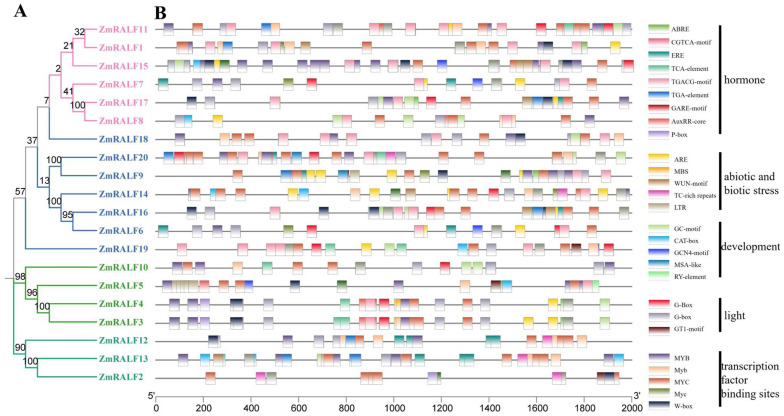
Analysis of cis-elements of *ZmRALF* gene promoter sequences. (**A**) This evolutionary tree was generated by MEGA 7.0 software (method: neighbor-joining; bootstrap: 1000). (**B**) Cis-elements in the 2 kb promoter sequences of *ZmRALF* genes were predicted. These cis-elements include hormone-related, abiotic and biotic-related, growth and development-related, light-related, and transcription factor binding sites-related.

To explore the potential functions and regulatory mechanisms of the *ZmRALF* gene family, we utilized the PlantCARE database to predict possible cis-elements within the 2000 bp promoter regions of each *ZmRALF* gene. As shown in Figure 7, we identified five primary types of cis-elements: hormone-related elements, growth and development-related elements, biotic and abiotic stress-related elements, and light-related elements. Specifically, the hormone-related elements include abscisic acid (ABA), auxin (IAA), methyl jasmonate (MeJA), and salicylic acid (SA). The growth and development-related elements encompass meristem expression, endosperm expression, seed specificity, and the cell cycle (Figure 7). The biotic and abiotic stress-related elements are associated with responses to drought, low temperature, wounding, and defense. Interestingly, we observed that most promoter regions of the *ZmRALF* genes contain elements related to abscisic acid, jasmonic acid, and drought stress-related elements. For example, *ZmRALF1*, *ZmRALF3*, *ZmRALF5*, *ZmRALF8*, *ZmRALF10, ZmRALF11*, *ZmRALF12*, *ZmRALF13*, *ZmRALF18*, and *ZmRALF20* suggest the involvement of *ZmRALF* genes in ABA, JA, and drought-signaling pathways (Figure 7). JA and SA pathways represent two major resistance-related signaling pathways in plants. Importantly, we found that the promoter regions of *ZmRALF11*, *ZmRALF15*, *ZmRALF18*, *ZmRALF19*, and *ZmRALF20* contain JA and SA cis-acting elements, suggesting that these genes may play a role in antagonistically regulating the JA and SA signaling pathways (Figure 7). These findings provide significant insights into the regulatory networks and potential functional roles of the *ZmRALF* gene family, highlighting their importance in hormone signaling and stress responses in plants.

### 2.7. Tissue-Specific Expression Analysis of ZmRALF

**Figure 8:**
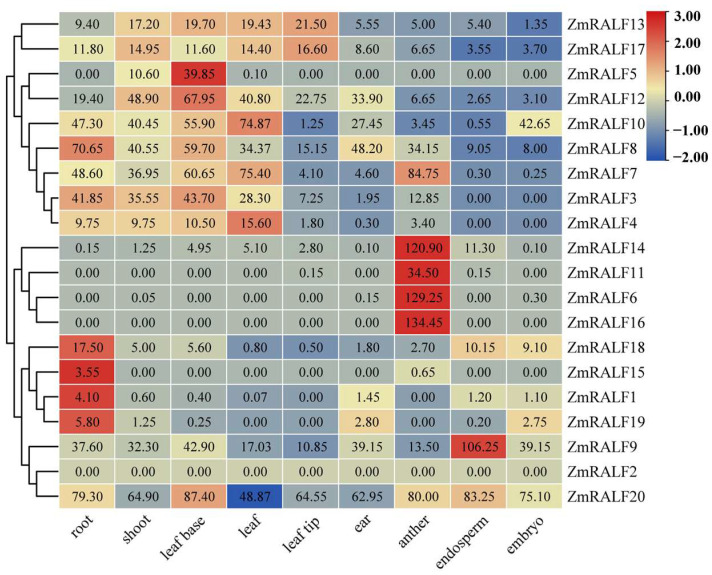
The tissue expression pattern of *ZmRALF* genes in different tissues including root, endosperm, leaf base, ear, embryo, anther, leaf tip, shoot, and leaf. Red and blue boxes indicate high and low expression levels of *ZmRALF* genes.

To further investigate the functions of the *ZmRALF* family genes, we examined their expression patterns in different tissues and organs using RNA-seq data. Our analysis revealed that *ZmRALF2* was not expressed in any of the tissues examined, suggesting that it may be a non-functional pseudogene. Alternatively, *ZmRALF2* might possess a specialized expression pattern that was not captured in the tissues we analyzed. Interestingly, several *ZmRALF* genes displayed tissue-specific expression patterns (Figure 8). For example, *ZmRALF6*, *ZmRALF11*, *ZmRALF14*, and *ZmRALF16* were highly expressed in anthers, while *ZmRALF1*, *ZmRALF15*, *ZmRALF18*, and *ZmRALF19* were predominantly expressed in roots (Figure 8). Conversely, *ZmRALF7*, *ZmRALF8*, *ZmRALF9*, *ZmRALF10*, *ZmRALF12*, *ZmRALF13*, *ZmRALF17*, *ZmRALF18*, and *ZmRALF20* exhibited widespread expression across various tissues and organs (Figure 8). These findings provide valuable insights into the diverse roles and regulatory mechanisms of *ZmRALF* genes, highlighting their tissue-specific and ubiquitous expression patterns.

### 2.8. Expression Patterns of ZmRALF under Abiotic Stresses

**Figure 9:**
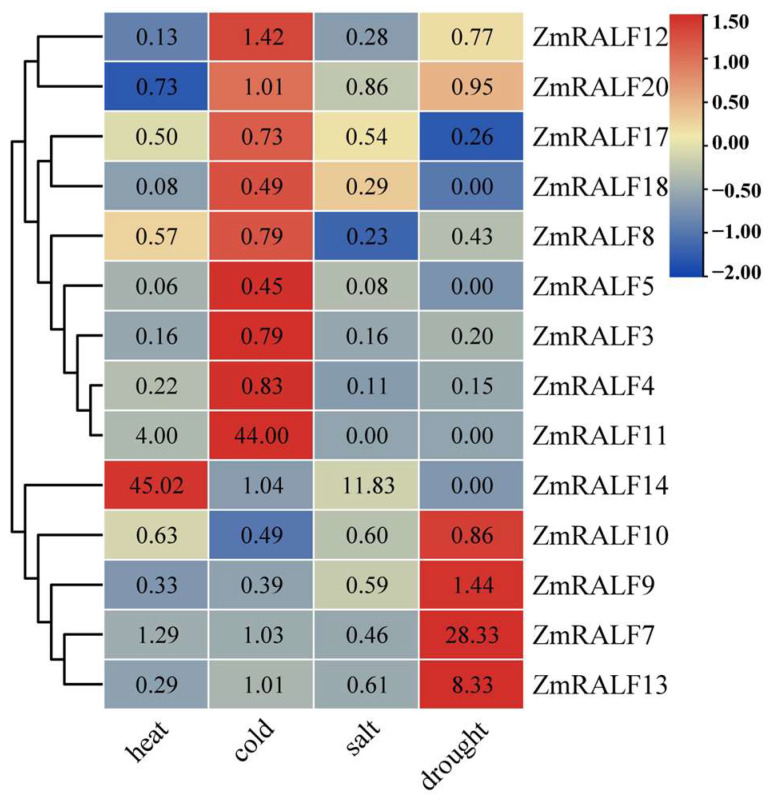
The expression pattern analysis of *ZmRALF* under different abiotic stresses including drought, heat, salt, and cold. Red and blue boxes indicate high and low expression levels of *ZmRALF* genes.

RALF proteins play a crucial role in regulating plant growth, development, and responses to environmental changes [24,25]. We investigated the expression patterns of *ZmRALF* genes under various abiotic stresses, including drought, heat, cold, and salt, using RNA-seq data. Fold change greater than 1.2 represents up-regulation, and fold change less than 0.7 represents down-regulation. Our analysis revealed that the expression patterns of *ZmRALF* genes varied under different stress conditions. Specifically, we observed significant up-regulation of 3, 2, 1, and 3 *ZmRALF* genes, respectively, and down-regulation of 10, 4, 11, and 4 genes, respectively, under heat, cold, salt, and drought stresses (Figure 9). For example, the expression levels of *ZmRALF7*, *ZmRALF9*, and *ZmRALF13* were significantly up-regulated under drought stress, while *ZmRALF7*, *ZmRALF11*, and *ZmRALF14* showed significant expression changes in response to heat stress. Interestingly, the expression levels of most *ZmRALF* genes were significantly down-regulated under salt stress, except *ZmRALF11* and *ZmRALF14*. Notably, some *ZmRALF* genes exhibited opposing expression patterns under different stresses. For instance, *ZmRALF9* and *ZmRALF13* were significantly up-regulated under drought stress but down-regulated under salt and heat stresses. Conversely, some *ZmRALF* genes displayed similar expression patterns across different stresses. For example, *ZmRALF3*, *ZmRALF4*, *ZmRALF12*, *ZmRALF17*, and *ZmRALF18* were down-regulated under drought, salt, and heat stresses. These findings provide valuable insights into the complex regulatory networks and stress response mechanisms of *ZmRALF* genes, highlighting their diverse roles and adaptive significance in maize.

### 2.9. Expression Analysis of ZmRALF Genes under NaCl and PEG Treatments by RT-qPCR

**Figure 10:**
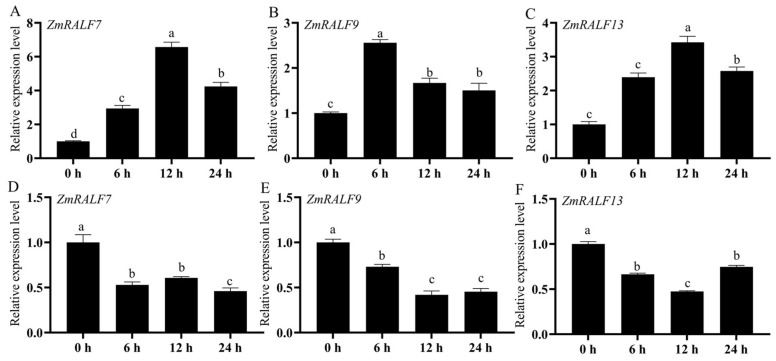
The relative expression patterns of *ZmRALF* genes under 20% PEG6000 and 200 mM NaCl stresses, as determined by RT-qPCR. (**A**–**C**) Expression levels of *ZmRALF7*, *ZmRALF9*, and *RALF13* genes in response to 20% PEG6000 treatment. (**D**–**F**) Expression levels of *ZmRALF7*, *ZmRALF9*, and *RALF13* genes in response to 200 mM NaCl treatment. The expression levels at subsequent time points were calculated relative to the 0 h measurement. The data include the standard error (SE) based on three replicates. Different letters indicate significant differences determined by one-way analysis of variance (ANOVA). RT-qPCR analysis utilized the *Zm00001d013367* gene as an internal control.

To verify the results of RNA-seq, we selected the ZmRALF7, ZmRALF9, and ZmRALF13 genes, which exhibited responsiveness to both drought and salt stress conditions. An RT-qPCR test was employed to assess the transcription levels of these genes following treatment with 200 mM NaCl and 20% PEG6000. Maize seedlings at the three-leaf stage were subjected to treatments with 200 mM NaCl and 20% PEG6000, and root samples were collected at 0 h, 6 h, 12 h, and 24 h. RNA was extracted from the roots and reverse-transcribed into cDNA. The results indicated that the expression levels of ZmRALF7, ZmRALF9, and ZmRALF13 were altered by NaCl stress (Figure 10). Notably, the expression levels of ZmRALF7, ZmRALF9, and ZmRALF13 were significantly up-regulated after 6 h of NaCl treatment; however, the extent of up-regulation varied among the ZmRALF genes. For instance, after 12 h of NaCl treatment, the expression of ZmRALF7 was up-regulated 7-fold, while the expression of ZmRALF9 was only up-regulated 1.7-fold. It is important to note that the expression levels of ZmRALF7 and ZmRALF13 began to decrease at 12 h post-NaCl treatment, whereas the expression of ZmRALF9 began to decline at 6 h. In contrast, under 20% PEG6000 treatment, the expression of ZmRALF7, ZmRALF9, and ZmRALF13 exhibited down-regulation with significant decreases observed at each time point during PEG treatment. These results suggest that certain ZmRALF genes play complex regulatory roles in the maize response to salt and drought stresses.

## 3. Discussion

RALF plays a key role in plant growth, development, and responses to abiotic and biotic stresses. However, our understanding of the potential functions of *ZmRALF* in maize remains limited. In this study, we identified 24 *RALF* members in the maize genome. Previous studies have reported the identification of 35, 43, 61, 24, 17, 12, and 18 RALF members in *Arabidopsis*, rice, *Brassica napus*, soybean, Lotus, Medicago, and quinoa, respectively [5,6,22,24]. The number of *ZmRALF* genes is lower than in *Arabidopsis*, *Brassica napus*, and rice but higher than in Lotus, Medicago, and quinoa. Several factors could account for these differences. For instance, *Brassica napus* is an allotetraploid species resulting from the hybridization of *B. oleracea* and *B. rapa*, which led to a doubling of its chromosome number, resulting in a significant increase in gene numbers [26]. Additionally, maize [27] has a larger genome compared to Lotus [28] and Medicago [29]. These findings indicate that the number of RALF members varies among different species, suggesting that *RALF* genes exhibit both functional conservation and functional differentiation across species.

Gene replication serves as a primary driving force behind species evolution, gene expansion, and the emergence of new gene functions. These replication events generate a wealth of genetic variation within gene families, facilitating their adaptation to environmental changes [30]. Among these duplication events, WGD and TD are crucial for gene family expansion and the creation of new genes [31]. Previous studies have demonstrated that TD is the major contributor to the expansion of the RALF gene family in both rice and *Arabidopsis* [5,32]. In contrast, the amplification of the *RALF* gene family in legume plants and strawberry primarily relies on WGD [18,22]. In maize, we identified six WGD gene pairs and two TD gene pairs. This suggests that WGD plays a predominant role in the expansion of the maize *RALF* gene family. Our findings indicate that the mode of expansion for the *RALF* gene family varies significantly across different species, which may also imply functional differences.

RALF is a type of cysteine-rich, plant-secreted peptide that undergoes precursor peptide processing and folding to form active mature peptides within the plant body. The full-length RALF precursor peptide ranges from approximately 80 to 150 amino acids with a non-conserved signal peptide region at the N-terminus [3] and an RRXL cleavage recognition site in the middle [33]. The mature peptide contains a conserved YISY motif at the N-terminus, which is critical for receptor binding [32], and four cysteine residues at the C-terminus [3]. RALF peptides regulate various biological processes by binding to cell membrane receptors [12]. CrRLK1Ls (Catharanthus roseus Receptor-like Kinases 1-Like) are receptor kinases widely present in plants [34]. FER, ANX1/2, BUPS1/2, and THE1 have been shown to interact with RALF peptides [35], enabling the sensing of environmental changes and the regulation of plant growth, development, and stress responses [35]. Notably, the N-terminus of mature RALF peptides contains the YISY/YIXY motif, which is essential for binding to the FER receptor. Studies have shown that the absence of the YISY motif diminishes the inhibitory effect of RALF on root elongation [10]. As illustrated in Figure 2, there are notable structural variations among RALF1/2/9/11/15/17/18/20, including the absence of RRXL cleavage sites and YISY motifs. In contrast, RALF3/5/8/12/13 peptides possess a relatively complete structure, including RRXL cleavage sites, YISY motifs, and four cysteine residues, suggesting that these RALFs may serve as potential ligands for CrRLK1L receptor kinase binding. In future research, we plan to perform whole-genome identification of CrRLK1L in maize and screen potential RALF ligands through fluorescence complementation experiments and Co-immunoprecipitation (Co-IP) screening.

Numerous *Arabidopsis* RALF peptides have been demonstrated to inhibit root development [36]. Additionally, the inhibitory effect of RALF on root growth is mediated through interactions with other hormones or reactive oxygen species (ROS) signaling. For instance, Li et al. through RT-qPCR and auxin metabolome analysis confirmed that the RALF1-FER complex functions as a positive regulatory factor within the IAA signaling pathway by activating the expression of the IAA biosynthesis gene YUCCA and the downstream transcription factors TIR1 and AFB, ultimately leading to the inhibition of root elongation [37]. Our findings indicate that *ZmRALF1/15/18/19* is highly expressed in roots (Figure 8), and the promoter region of *ZmRALF1* contains IAA-responsive elements. This suggests that the *ZmRALF1* gene may modulate the IAA signaling pathway by activating the expression of the IAA biosynthesis gene to influence root development. Notably, *ZmRALF1* and *AtRALF1* belong to the same subgroup, which supports this inference (Figure 3). This presents an intriguing narrative, and we plan to verify our hypothesis through auxin metabolome experiments in the future.

Plant peptides can activate hormone signaling pathways such as auxin, ethylene, and abscisic acid, thereby regulating the expression of stress-responsive genes mediated by secondary signals, including Ca^2+^ and MAPK [38]. For example, the *CLE9* peptide regulates stomatal pore size in an ABA-dependent manner and activates the MAPK signaling cascade by binding to the BAM1/3 receptor, thereby enhancing the drought tolerance of Arabidopsis thaliana [39]. The CEP5 peptide contributes to the drought tolerance regulation of Arabidopsis thaliana by binding to the CEPR1/XIP1 receptor and inhibiting the expression of genes associated with down-regulating auxin synthesis [40]. The RALF22/23 small peptide binds to the FER receptor, inhibits ABA signaling, promotes ROS accumulation in Arabidopsis, and plays a role in the salt stress response [16]. In our study, we observed that the expression levels of *ZmRALF7*, *ZmRALF9*, and *ZmRALF13* genes were up-regulated and down-regulated under PEG and NaCl treatment, (Figure 10). Moreover, we found that the promoter region of the *ZmRALF7*, *ZmRALF9*, and *ZmRALF13* genes contains drought and ABA response elements. The rice malectin-like receptor protein kinase OsMRLK63 regulates drought tolerance in rice by modulating intracellular ROS levels through the OsRALF45/46-OsMRLK63-OsRboh signaling module [25]. Interestingly, the proteins sequences of *ZmRALF7*, *ZmRALF9*, and *ZmRALF13* contain a conserved YISY motif, which is essential for the binding of RALF to its receptor FER (Figure 2), indicating that ZmRALF7, ZmRALF9, and the ZmRALF13-ZmCrRLK1L signaling module may enhance drought tolerance by modulating intracellular ROS levels in maize. Future studies should encompass phenotypic assessments to fortify our understanding of the precise mechanisms by which *ZmRALF* family members contribute to the intricate process of drought and salt stresses in maize.

The process of double fertilization in plants encompasses a series of complex biological events [41], in which RALF peptides play significant regulatory roles in various signal transduction pathways in double fertilization. For instance, the small peptide AtRALF33 promotes the production of reactive oxygen species (ROS) and inhibits pollen hydration in the stigma via the FER/ANJ-LLG1-ROP2-RBOHD signaling pathway [42]. Conversely, the small peptide PCP-Bγ can compete with AtRALF33 for binding to FER, thereby reducing ROS accumulation in cells and promoting pollen–stigma recognition [43]. AtRALF4 and AtRALF19 serve as ligands for FER, ANX1/2, and BUPS1/2, playing crucial roles in regulating pollen tube growth and maintaining the structural integrity of pollen tube cell walls [8]. As shown in Figure 7, *ZmRALF6/11/14/16* is highly expressed in anthers. We conclude that ZmRALF6/11/14/16 may function as a ligand for ZmCrRLK1L, forming the ZmRALFR6/11/14/16-ZmCrRLK1L complex. ZmRALFR6/11/14/16 promotes the production of ROS and inhibits pollen hydration in the stigma via the ZmCrRLK1L-RBOHD signaling pathway, which regulates pollen tube growth and maintains the integrity of the pollen tube cell wall structure. However, it is imperative to acknowledge that this conclusion is limited. Future studies should encompass phenotypic assessments to fortify our understanding of the precise mechanisms by which *ZmRALF* and *ZmCrRLK1L* family members contribute to the intricate process of pollen tube growth in maize.

## 4. Materials and Methods

### 4.1. Identification and Phylogenetics of ZmRALF Family Genes in Maize

The whole-genome sequence and genome annotation file of maize were downloaded from Phytozome 13 (https://phytozome-next.jgi.doe.gov/, accessed on 15 July 2024) [44]. The Hidden Markov Model (HMM) for the RALF domain (PF05498) was obtained from the Pfam database (http://pfam-legacy.xfam.org/, accessed on 15 July 2024) [45]. Thirty-seven AtRALF protein sequences were downloaded from TAIR database (http://www.arabidopsis.org/, accessed on 15 July 2024) [46]. We utilized the whole-genome sequence and genome annotation file to extract the coding sequences (CDS) of maize, obtaining the longest transcript using the R package seqfinder (https://github.com/yueliu1115/seqfinder, accessed on 15 July 2024), which was then converted into protein sequences. Firstly, we employed HMM software to search for ZmRALF in the maize protein database (E-value < 1 × 10^−5^). Secondly, the BLASTP method was used with the AtRALF protein sequence and maize protein sequence (E-value < 1 × 10^−5^). Then, candidate ZmRALF protein sequences were submitted to the NCBI-CDD (https://www.ncbi.nlm.nih.gov/Structure/bwrpsb/bwrpsb.cgi, accessed on 16 July 2024) to identify the conserved RALF domain.

The phylogenetic tree was constructed from ZmRALF, OsEALF, SiRALF, SbRALF, and AtRALF protein sequences based on MEGA 7.0 software employing the neighbor-joining (N-J) method with a bootstrap number of 1000 [47].

### 4.2. Analysis of ZmRALF Gene Structures, Protein Motifs, and Cis-Acting Elements 

The gene structures and protein conserved motifs of ZmRALF were identified through genome annotation files and MEME (motif length: use built-in parameters (6–60 aa); motif number: 10) [48], respectively. The 2 kb promoter sequence of *ZmRALF* genes was submitted to PlantCARE (http://bioinformatics.psb.ugent.be/webtools/plantcare/html/, accessed on 15 July 2024) to predict potential cis-acting elements [49], then the picture of a phylogenetic tree and cis-acting elements was visualized based on Tbtools v2.121 (Beijing, China) software [50].

### 4.3. Homologous Gene Pair and ka/ks Ratio Analysis of ZmRALF

Tandem duplication (TD) and whole-genome duplication (WGD) events analysis were performed by MCscan software (using built-in parameters). The ka, ks, and ka/ks were calculated by Tbtools v2.121 (Beijing, China) software. The collinear gene pairs between *ZmRALF* and *OsRALF* and *AtRALF* were analyzed by MCscan software (using built-in parameters) [51].

### 4.4. ZmRALF Gene Expression Patterns Analysis

The tissue expression profiles of the *ZmRALF* gene from various tissues were obtained from the PPRD database [52] (PRJEB35943). The maize breed examined was the B73 inbred line; the tissues analyzed included embryo, endosperm, anther, ear, leaf tip, leaf, leaf base, shoot, and root (Supplemental Table S4). The RNA-seq data was from maize under different abiotic stresses, such as drought (PRJNA378714), salt (PRJNA244661), cold (PRJNA244661), and heat (PRJNA244661) (Supplemental Table S5). The gene expression was quantified using FPKM values. The expression change was calculated as (1 + FPKM in the treatment group) / (1 + FPKM in the control group), where a fold change greater than 1.2 indicates up-regulation, and a fold change less than 0.7 indicates down-regulation. Heat maps were generated using Tbtools v2.121 (Beijing, China) software.

### 4.5. Plant Materials, Growth Conditions, and PEG and NaCl Treatments

The maize cultivar B73 inbred line was utilized in this study. The maize seeds were disinfected with 75% ethanol for 1 min, and subsequently washed five times with distilled water to eliminate any residual ethanol. The sterilized seeds were evenly distributed in seedling pots, covered with vermiculite, and irrigated with distilled water to ensure adequate moisture absorption by the vermiculite [53]. After three days of germination, the seeds were transferred to black boxes containing Hoagland nutrient solution [53], which was replaced every three days. The maize seedlings were cultivated in a greenhouse under conditions of 14 h of light and 10 h of darkness (200 µmol m^−2^ s^−1^) at a temperature of 27 °C.

For the 20% PEG6000 and 200 mM NaCl treatments, the maize seedlings were cultured in Hoagland nutrient solution until reaching the three-leaf stage, after which they were transferred into normal solution, 20% PEG6000, and 200 mM NaCl, respectively [53]. Roots samples were collected at 0 h, 6 h, 12 h, and 24 h following the initiation of the stress treatment [53]. The concentrations used and the sampling time points were based on the methodology described by Zhu et al. [53].

### 4.6. Total RNA Extraction and RT-qPCR Analysis

In our study, we utilized Primer 5.0 software to design *ZmRALF* gene-specific RT-qPCR primers (Supplementary Table S6). Total RNA was extracted from roots using the E.Z.N.A.^®^ Plant RNA Ki. cDNA was synthesized through reverse transcription using the PrimeScript™ RT reagent Kit. The RT-qPCR assays were conducted with a real-time PCR analyzer (CFX384 Touch Real-Time PCR Detection System) (Bio-Rad, Hercules, CA, USA). The RT-qPCR reaction mixture comprised  5 µL SYBR, 3 µL No RNA enzyme water, 1 µL cDNA, 0.5 µL forward primer, and 0.5 µL reverse primer, resulting in a final volume of 10 µL. The RT-qPCR amplification program consisted of an initial step at 95 °C for 5 min, followed by 45 cycles of 95 °C for 15 s and 60 °C for 1 min.

### 4.7. Statistical Analysis

The relative expression levels of *ZmRALF* genes were calculated using the 2^−ΔΔCt^ method. The data are expressed as the standard error (SE) based on three replicates. Different letters indicate significant differences by one-way analysis of variance (ANOVA). The gene expression levels at 0 h were set to 1, with relative expression levels at other time points calculated relative to 0 h. RT-qPCR analysis was conducted using the *Zm00001d013367* gene as an internal control.

## 5. Conclusions

In this study, we conducted a genome-wide identification of *RALF* genes in maize and performed a comprehensive analysis of their physicochemical properties, phylogenetic relationships, conserved motifs, gene structures, gene duplication events, and tissue expression patterns. We also characterized the expression profiles of *ZmRALF* genes following different abiotic treatments. Notably, *ZmRALF1/15/18/19* is highly expressed in roots, while another member *ZmRALF6/11/14/16* exhibits elevated expression in anthers. The expression levels of *ZmRALF7/11/13/14* genes are significantly influenced by drought, salt, cold, and heat, indicating their potential role in response to abiotic stresses. Our findings provide new insights into the function of *RALF* genes in maize.

## Data Availability

All data, materials, software applications, and custom code supporting the claims made in this article are in full compliance with field standards. Data are contained within the article or Supplementary Material. The datasets of public RNA-seq data from the current study are available in the PPRD (http://ipf.sustech.edu.cn/pub/plantrna/, accessed on 16 July 2024).

## Supplementary Materials

The following supporting information can be downloaded at https://www.mdpi.com/article/10.3390/plants13202883/s1: Supplemental Table S1. Physicochemical properties of *ZmRALF* family gene in maize. Supplemental Table S2. Ka/Ks values of duplicate gene pairs in maize. Supplemental Table S3. The cis-element analysis of *ZmRALF* genes in maize. Supplemental Table S4. The tissue expression patterns of *ZmRALF* genes in maize. Supplemental Table S5. The expression patterns of *ZmRALF* genes of abiotic stresses in maize. Supplemental Table S6. The primer sequences of *ZmRALF* genes used for RT-qPCR.
